# Visualizing vastness: Graphical methods for multiverse analysis

**DOI:** 10.1371/journal.pone.0339452

**Published:** 2026-02-05

**Authors:** Daniel Krähmer, Cristobal Young

**Affiliations:** 1 Department of Sociology, University of Munich, Munich, Germany; 2 Department of Sociology, Cornell University, Ithaca, New York, United States of America; German Institute for Adult Education - Leibniz Center for Lifelong Learning, GERMANY

## Abstract

Multiverse analysis is an increasingly popular tool for improving the robustness and transparency of empirical research. Yet, visualization techniques for multiverse analysis are underdeveloped. We identify critical weaknesses in existing multiverse visualizations—specification curves and density plots—and introduce a novel alternative: multiverse plots. Using both simulated and real-world data, we illustrate how multiverse plots can retain detailed information even in the face of thousands of model specifications. Multiverse plots overcome key limitations of existing methods by eliminating arbitrary sampling (a common issue with specification curves) and information loss on analytical decisions (an issue with density plots). Furthermore, they effectively show what conclusions a dataset can reasonably support and which researcher decisions drive variation in results. By providing software code to generate multiverse plots in Stata and R, we enable analysts to visualize multiverse results transparently and comprehensively.

## Introduction

Amid concerns about the replicability, substance, and credibility of science, multiverse analysis has been heralded as a method to improve the robustness and transparency of research. The idea behind the approach—variously referred to as “multiverse analysis” [1], “multimodel analysis” [2], “specification curve analysis” [3], or “vibration of effects” [4]—is straightforward. Since there are many credible ways of formulating a statistical analysis, and any single estimate may suffer from selective reporting, multiverse analysis explores all reasonable model specifications. It contrasts the author’s preferred estimate with the range of possible estimates, providing a broader, more transparent understanding of the data and decisions made in the analysis. Instead of drawing readers into a dark corner of the “garden of forking paths” [5], multiverse analysis provides a bird’s-eye view of the maze of researcher decisions and the resulting range of defensible findings.

Multiverse analysis begins by identifying a general target estimand—such as the effect of gender on wages—and proceeds to estimate all plausible models to identify this effect. Instead of yielding a single point estimate, multiverse analysis generates many estimates, each corresponding to a different model specification. Each model represents a reasonable combination of analytical decisions, such as including or excluding a certain control variable, using a different functional form, or handling missing data differently.

Defining what counts as a ‘reasonable’ model specification is one of the most intellectually engaging aspects of multiverse analysis. The approach encourages researchers to thoughtfully map the space of defensible analytic choices—grounded in theory, data structure, and methodological standards, fostering a broader, more rigorous consideration of plausible specifications. When well-defined, the multiverse sharpens our understanding of how analytical decisions shape substantive conclusions—especially in cases where no single specification is clearly superior. In such situations, multiverse analysis becomes not just useful but essential, offering a principled framework for confronting model uncertainty head-on (for details on defining the multiverse, see [6–9]).

Although multiverse analysis has been successfully applied in psychology [10–12], epidemiology [13,14], medicine [15], sociology [16], education research [17], and political science [18], a practical challenge remains: multiverse results are inherently difficult to visualize, which can limit their interpretability and impact. The key challenge is that multiverse results expand exponentially as the number of analytical decisions increases. With just four binary decisions (e.g., whether to include or exclude a control variable, retain or remove an outlier, delete or impute missing values, apply or ignore weights), the multiverse contains 2^4^, or 16, possible models. With 10 analytical choices, this number balloons to 2^10^ = 1,024 models, and with 20 choices, it exceeds one million models. The largest multiverse analysis to date estimated a staggering 9 billion regressions [19].

To effectively communicate this wealth of information, researchers require graphical methods to present multiverse results. In this contribution, we review two existing multiverse visualizations—specification curves and density plots—and highlight their strengths and limitations. Building on this review, we introduce multiverse plots, a novel graphical method that integrates the key advantages of existing approaches. Multiverse plots combine a density-based summary of the modeling distribution with a binned decision dashboard on analytical choices, allowing researchers to present the results from many model specifications while also revealing which analytical choices most influence the results. Using both simulated and real-world data, we demonstrate the practical advantages of multiverse plots. By providing software code to generate multiverse plots in Stata and R, we enable multiverse analysts to communicate their results transparently and comprehensively.

## Visualizing many estimates: Specification curves vs. density plots

Multiverse visualizations come in various forms [20], but typically fall into one of two broad categories: specification curves [3] and density plots [2]. To illustrate both approaches, we use simulated data from a hypothetical multiverse in which researchers face ten decisions, each with two defensible options (X or Y), resulting in 1,024 unique estimates.

**Fig 1:**
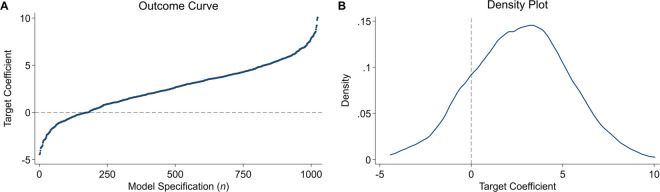
Multiverse visualizations: Outcome curves vs. density plots. Both graphs present results from a simulated multiverse with 10 binary researcher decisions, i.e., 1024 (2^10^) unique estimates. Panel A shows the cumulative graph with point estimates on the y axis, sorted in ascending order. Panel B shows the density plot, with point estimates on the x axis.

Specification curves, in their stripped-down form known as “outcome curves” [20], arrange multiverse estimates by the size of the target coefficient and plot them in ascending order. This yields a cumulative distribution (Fig 1A). Density plots, by contrast, follow standard conventions for visualizing sampling distributions. They show how frequently different effect sizes appear across the model space (Fig 1B). Both visualizations convey the same information—only the axes are flipped: while outcome curves plot effect sizes on the y-axis, density plots place them on the x-axis. For researchers trained in statistical inference, the x-axis layout of density plots often feels more intuitive, aligning with how sampling distributions are typically presented [21,22]. That said, this distinction may ultimately come down to visual style and disciplinary habit.

In their basic form, both graphs display two pieces of information: the range of possible estimates and their concentration. Fig 1 shows that point estimates in our simulated multiverse range from –5 to +10, with most estimates falling between 0 and +5. This indicates that although the multiverse generally shows (moderately) positive results, researchers may still find a negative or null effect depending on their model specification. In such cases, where results vary widely, merely diagnosing a lack of robustness is often unsatisfying. Analysts typically want to understand *why* results vary, i.e., which decisions drive estimates higher or lower. This is where specification curves come into play. They supplement outcome curves with a decision dashboard that displays, using a dot matrix, the analytical choices underlying each point estimate.

**Fig 2:**
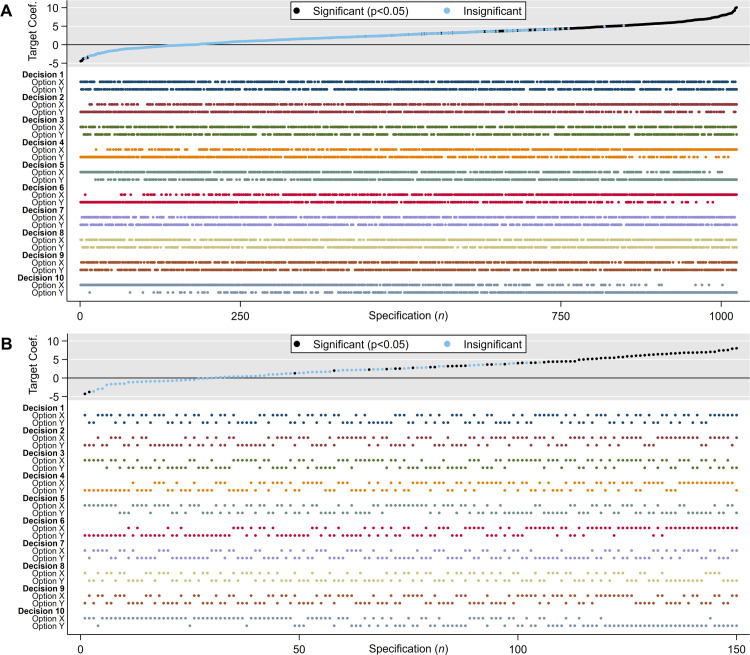
Specification curves. Specification curves for a multiverse of 10 binary researcher decisions. Panel A plots all 1024 (2^10^) model specifications, Panel B plots a random subset (n = 150). Panel A shows the full outcome curve but sacrifices readability in the decision dashboard. Panel B fails to capture the full range of possible estimates.

Fig 2A displays a specification curve. The graph’s top panel revisits the familiar outcome curve from Fig 1A—now also distinguishing statistical significance—while the graph’s colored bottom panel contains a decision dashboard. This dashboard marks, for every researcher decision, which analytical option (X or Y) underlies the point estimate presented above. As proponents of specification curves argue, this presentation allows analysts and readers to identify influential researcher decisions. If dots in the bottom panel discernibly cluster or disperse—so the simple visual heuristic goes—the corresponding researcher decision is influential as it shifts point estimates left or right, leading to systematically smaller or larger results.

In practice, specification curves face a fundamental limitation: they do not scale well, undermining their ability to convey model influence in large multiverses. While the number of multiverse specifications grows exponentially, specification curves do not scale accordingly. They typically max out at around 200 to 300 estimates before the decision dashboard collapses into visual “fuzz.” This is evident in Fig 2A, where the dots have blurred into near-continuous lines, with only slight variation at the extremes. Once specification curves become too compressed to interpret, they lose their core advantage: the ability to reveal which analytical choices drive variation in results.

Simonsohn *et al*. [3], who introduced specification curves, recognized this problem and suggested to plot only a sample of model specifications. But sampling introduces its own complications, and the cure can be worse than the disease. Conceptually, sampling undercuts the core premise of multiverse analysis: to consider the full range of defensible results. In practice, it inevitably distorts at least one important aspect of the modeling distribution.

The original specification curve article—despite recommending reporting a “random subset” of specifications [3]—used a non-random sampling strategy: presenting the 50 largest and smallest estimates of a multiverse of 1,728 analyses along with an additional 200 randomly chosen ones. This approach sharply overrepresents the tails of the distribution while excluding the moderate middle—only 12% of the mid-range estimates (200 of 1,628) are included. In large multiverses, this kind of non-random sampling can sharply transform multiverse results, skewing evidence more profoundly than even the original modeling assumptions could do (see S1 File).

Random sampling, by contrast, introduces uncertainty about whether the selected specifications adequately represent the full multiverse—particularly the extreme bounds, which are often a focal point of multiverse analysis. Long-tailed distributions, where researcher discretion is most consequential, are especially prone to underrepresentation. Fig 2B illustrates this risk. It displays 150 randomly sampled specifications (14.6% of the full multiverse), improving the readability of the decision dashboard but missing the full modeling range. The largest estimate shown is +8, while the true maximum is +10. This gap points to a broader limitation of random sampling: it can truncate the observed distribution and mislead readers about what conclusions the data might reasonably support. This is especially consequential for extreme bounds analysis, a classic multiverse approach that highlights the largest and smallest defensible estimates [23]. Because these extremes are rare, they are highly sensitive to sampling and may be excluded entirely by chance. The likelihood of missing them rises sharply as the multiverse expands and as the extremes become more isolated—precisely when model uncertainty is most consequential and most in need of visibility.

In short, once sampling is introduced—whether random or selective—preserving a complete and faithful view of the modeling distribution is difficult [24]. Random sampling risks omitting the extreme bounds, while non-random sampling can distort the overall shape of the distribution. Seen in this light, Simonsohn *et al*.’s choice of a non-random sampling strategy is understandable: it preserves the limiting-case extreme results. But this comes at a cost—namely, a systematic skewing of other regions of the distribution that are no less important for interpreting model uncertainty.

Density plots, unlike specification curves, avoid this scalability problem. Because their physical dimensions are not tied to the number of specifications, they can effortlessly visualize millions of estimates without sampling. However, while density plots scale well, they do not address a central question: which modeling decisions drive variation in estimates? To date, density plots have not been paired with a decision dashboard, leaving analysts with no visual representation of how specific analytical choices shape the distribution. As a result, multiverse analysts face an interpretive tradeoff: use specification curves and risk distortion through sampling, or use density plots and lose visual insight into model influence.

To resolve this dilemma, we introduce multiverse plots: a new graphical method that preserves the interpretive power of specification curves while adopting the scalability and clarity of density plots. Multiverse plots retain a dashboard, enabling analysts to visualize which modeling decisions drive variation in results. But instead of relying on a cumulative distribution (as in specification curves), they pair the dashboard with a density-based summary of the full modeling distribution—eliminating the need for sampling and allowing thousands or even millions of specifications to be visualized without loss of clarity.

## The best of both worlds: Multiverse plots

**Fig 3:**
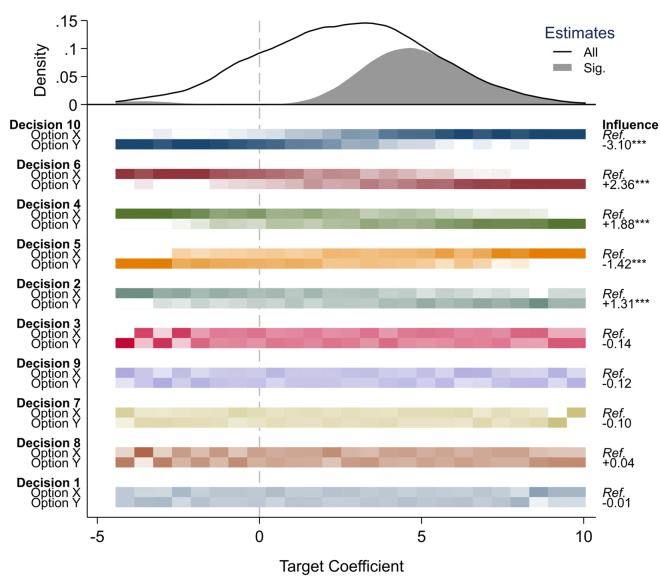
Multiverse plot. Multiverse plot based on 1,024 model specifications. The top panel shows the distribution of the target coefficient, separated by statistical significance (*p* < .05). The bottom panel cuts the modeling distribution into 25 segments, with darker color shades representing higher relative frequencies of analytical choices. Influence estimates on the right come from a weighted-least-squares meta-regression quantifying how much the target coefficient changes upon switching from Option X to Option Y.   *p* < .05;    *p* < .01;     *p* < .001.

Fig 3 introduces the multiverse plot. The graph’s top panel displays a smoothed density of 1,024 model estimates for the target coefficient. The blue line shows the full distribution, while the shaded maroon area highlights the subset of statistically significant estimates (*p* < .05). The plot reminds us that estimates in the simulated multiverse span from –5 to +10, with most falling between 0 and +5, indicating a tendency toward moderately positive results. However, a small number of significant estimates appear at the far left of the distribution, suggesting that even strongly negative results are possible under certain modeling choices. While only few specifications produce significant negative estimates, these are qualitatively important. They imply that if a researcher is convinced the result must be negative, they will be able to find a few specifications in the model space that confirm their expectation—even though these are knife-edge specifications [23]. Overall, the upper panel of the multiverse plot suggests mixed robustness. Of all 1,024 model specifications, about half (42%) yield a significant positive effect, more than half (57%) show no significant effect, and very few (1%) produce a significant negative effect.

What sets multiverse plots apart from density curves is the bottom panel: the decision dashboard. This panel displays how each modeling decision shapes the distribution of estimates. Each row corresponds to a binary decision (Option X vs. Option Y), and each vertical segment aggregates models with similar target estimates. Within each segment, cells are shaded to show the relative frequency of that option—darker shades indicate that a given decision was more common among models producing estimates in that range.

To assess model influence, readers scan horizontally across each row. A visible gradient—where shading intensifies or fades consistently from left to right—signals that a particular decision is systematically associated with larger or smaller effects. For example, in the top row-pair (Decision 10), we see a stark gradient: Option Y is more common among the leftmost (negative) estimates, while Option X dominates the right. This suggests that switching from X to Y leads to smaller effect sizes.

The right side of the plot formalizes this interpretation through influence coefficients, derived from a meta-regression analysis (MRA) [25]. This approach regresses the target coefficient on the analytic features of each model, estimating the average change in outcome when switching between Options X and Y. For Decision 10, the coefficient indicates that choosing Option Y instead of X decreases the target estimate by 3.10 units—the largest influence observed. Four other decisions—6, 4, 5, and 2—also show statistically significant effects, with shifts ranging from –1.42 to +2.36 units.

Notably, the influence regression sometimes reveals effects not obvious to the eye. Decision 2, for example, shows no clear shading pattern in the dashboard but has a statistically significant coefficient of +1.31. This illustrates the complementary value of combining visual and quantitative diagnostics: where one may be ambiguous, the other can provide confirmation.

A major advantage of multiverse plots over specification curves is their scalability. Because the dashboard reflects the relative composition of estimate bins—rather than plotting one column per model—it remains readable even as the multiverse grows into the thousands or millions of specifications. This makes it possible to explore model influence without sampling or sacrificing interpretability.

Finally, multiverse plots improve on prior influence frameworks by offering statistical benchmarks for decision importance. Whereas earlier approaches relied on informal judgments (e.g., whether a decision “seemed influential”), multiverse plots use meta-regression standard errors to test whether observed shifts are larger than expected by chance. This allows researchers to identify not just which decisions matter, but how much, and with what level of certainty.

In sum, the multiverse plot in Fig 3 communicates the full landscape of modeling uncertainty: it shows the distribution of possible results, highlights which are statistically significant, and reveals which modeling decisions are driving those results. By combining the interpretive power of specification curves with the scalability and clarity of density plots, multiverse plots offer a robust and comprehensive tool for visualizing analytical flexibility.

We now turn to a real-world example to illustrate how these advantages translate to applied research.

## The deadliness of female-named hurricanes

Are hurricanes with female names deadlier than hurricanes with male names? A high-profile study by Jung *et al*. [26] claimed to have documented such “implicit sexism,” suggesting that female-named hurricanes may appear less threatening to the public, leading to fewer precautions and higher death tolls. Numerous scholars questioned the credibility of this sensational finding, downloading and analyzing the data themselves, and proposing alternative model specifications that yielded different results [27–30]. Alternative specifications concerned the handling of outliers (3 options), the choice of regression model (2 options), the treatment of leverage points (4 options), the specification of interactions (6 options), the selection of control variables (3 options), the measurement of femininity (2 options), and the functional form of damages (2 options). Considering all possible combinations from these inputs yields a multiverse of 1,728 unique specifications [3,19].

**Fig 4:**
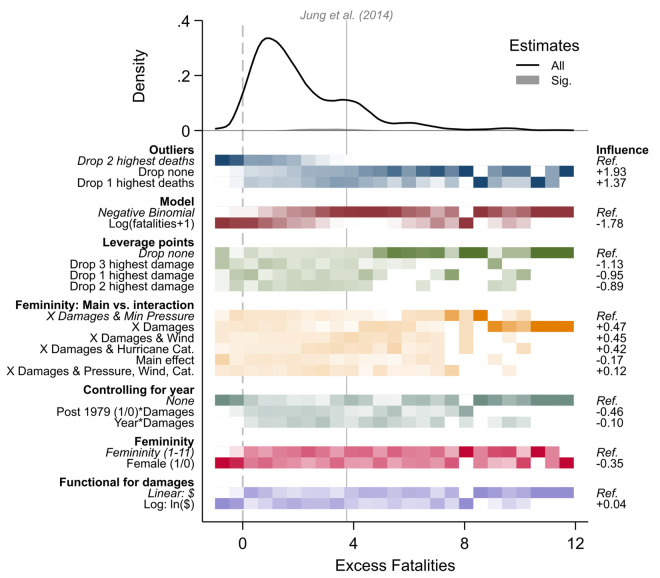
Multiverse plot of hurricane fatalities. Multiverse plot based on 1,728 model specifications [3], of which 1,704 (98.7%) yield a positive estimate, including 37 (2.1%) significant ones. Influence estimates on the right come from an OLS meta regression. The statistical significance of these influence coefficients is not reported.

Fig 4 presents the multiverse plot for the hurricane analysis, with the target coefficient being the estimated number of excess deaths attributable to a hurricane’s female (vs. male) name. The density curve in the top panel shows that almost all specifications yield positive estimates—most modestly, as indicated by the central hump near zero, and a few quite strongly, producing a long right tail. At first glance, this suggests that female-named hurricanes might be deadlier. However, the maroon shading reveals that almost none of these results are statistically significant. Of the 1,728 model specifications, 1,704 (98.7%) produce positive estimates, but only 37 (2.1%) reach significance at the 5% level. The original estimate from Jung *et al*. [26] lies near the upper extreme of the distribution, and is among the few 2 percent of estimates that achieve statistical significance. Although neither an exposed position in the modeling distribution nor an unusual *p*-value make an estimate less “true,” these findings raise concerns about the robustness of the original result. There are many reasonable ways of estimating the number of deaths associated with the gender of a hurricane’s name, and the vast majority of them are smaller and/or not statistically significant compared to the findings reported by the original authors.

To assess influence, consider the bottom panel of Fig 4. To the left of the graph, all researcher decisions (in bold) and their analytical options are listed. Options chosen by Jung *et al*. [26] are italicized and listed first so that influence coefficients on the right represent the average change in the target estimate upon deviating from the authors’ original specification. Estimates in this plot come from an OLS regression and do not include tests of significance (for a justification, see S1 File). Both the shading and influence coefficients highlight that the handling of outliers, the choice of regression model, and the treatment of leverage points are most influential. For outliers, alternatives to the original analytical strategy yield point estimates that are on average 1.37 to 1.93 units larger. For the regression model and leverage points, deviating from the original analytical strategy produces estimates that are between 0.89 and 1.78 units smaller. These influence effects are sizeable, given that most model specifications estimate the target coefficient to be between 0 and +4, and together they have the potential to shrink the original estimate of Jung *et al*. toward a null effect.

Overall, Fig 4 supports two key conclusions. The first is substantive: there is little systematic evidence that female-named hurricanes are deadlier than male-named ones. While the original specification by Jung *et al*. shows a significant effect, the overwhelming majority of plausible alternative specifications do not. This does not mean the original model is wrong—but it now stands nearly alone. Justifying it requires not only strong reasons for choosing that specification, but equally strong reasons for rejecting 98 percent of the modeling distribution. The finding rests on exceptionally narrow methodological ground: it holds only if every detail of the model is exactly right—and collapses if almost any detail is reconsidered. The second conclusion is methodological: when justifying a preferred model, researchers should focus on decisions that actually shape the results. Some modeling choices—like whether to measure femininity as a binary or continuous variable—may raise interesting methodological debates but have little bearing on the outcome. Others—such as how outliers are treated or which model type is used—clearly matter. For researchers interested in the substantive question, these are the decisions that deserve attention. In this way, multiverse plots serve a dual purpose: they communicate robustness while also focusing scholarly debate on the choices that truly influence the results.

## Conclusion and discussion

Multiverse analysis is a powerful tool for improving the transparency and robustness of empirical research, revealing both the range of results the data can support and how much discretion researchers have in selecting preferred results [1,9]. Yet despite its promise, the method faces a critical bottleneck: visualizing the multiverse in a way that is both scalable and interpretable. Existing approaches—like specification curves and density plots—each solve part of the problem but fall short when multiverses grow large or when the influence of modeling decisions needs to be assessed clearly.

In this paper, we introduced multiverse plots as a scalable, information-rich alternative that resolves these limitations. Multiverse plots preserve the interpretive strength of specification curves by retaining a decision dashboard, but avoid the combinatorial overload by pairing it with a density-based summary of the full modeling distribution. They scale seamlessly to thousands or even millions of model specifications without relying on sampling methods that inevitably skew either the shape of the modeling distribution or its extreme bounds. Crucially, multiverse plots supplement visual patterns with meta-regression–based influence estimates, providing statistical tests of which researcher decisions materially shape the results.

Compared to density plots, multiverse plots offer critical additional information: they do not just plot the shape of the distribution, but also its composition, the significance of results, and the specific modeling choices that matter. In this way, they not only communicate the robustness of findings, but also direct scholarly attention to the most consequential modeling assumptions.

Of course, multiverse plots do not preserve the full resolution of the data. They rely on binning the modeling distribution, which can obscure detail. Researchers concerned about this can increase the number of bins for a finer-grained summary; in the limiting case when the number of bins equals the number of specifications, the decision dashboards of multiverse plots converge back to those of specification curves. This flexibility means multiverse plots provide the most added value in large multiverses, where traditional methods become infeasible.

We provide open-source code in both Stata and R to make multiverse plots easy to implement and widely adoptable in applied research.

## Supporting information

S1 File Appendix. (PDF)
