# Supplementary material for: Visualizing vastness: Graphical methods for multiverse analysis
Source: PLoS One. 2026 Feb 5;21(2):e0339452. doi: 10.1371/journal.pone.0339452 (PMC12875576; doi:10.1371/journal.pone.0339452)
Supplement: S1 File — (PDF) [file pone.0339452.s001.pdf]

Visualizing vastness: Graphical methods for multiverse analysis
— Supporting Information —

Daniel Krähmer, Cristobal Young

Contents

Appendix A – Distortion of Multiverse Results Due to Non-Random Sampling 2
Appendix B – Influence regressions: WLS vs. OLS 3

Appendix A. Distortion of Multiverse Results Due to Non-Random Sampling.

Non-random sampling can distort the visual representation of multiverse results. To illustrate this problem, we simulated normally distributed multiverses of varying size (n = 500, 1000, 2000) and variance (σ = 1, 1.5, 2). Figure 1 overlays for each multiverse the distribution of all estimates with the distribution of a non-random subset, including the 50 largest, 50 smallest, and 200 randomly selected estimates. Panel A plots these multiverses as cumulative outcomes curves, Panel B plots them as density distributions.

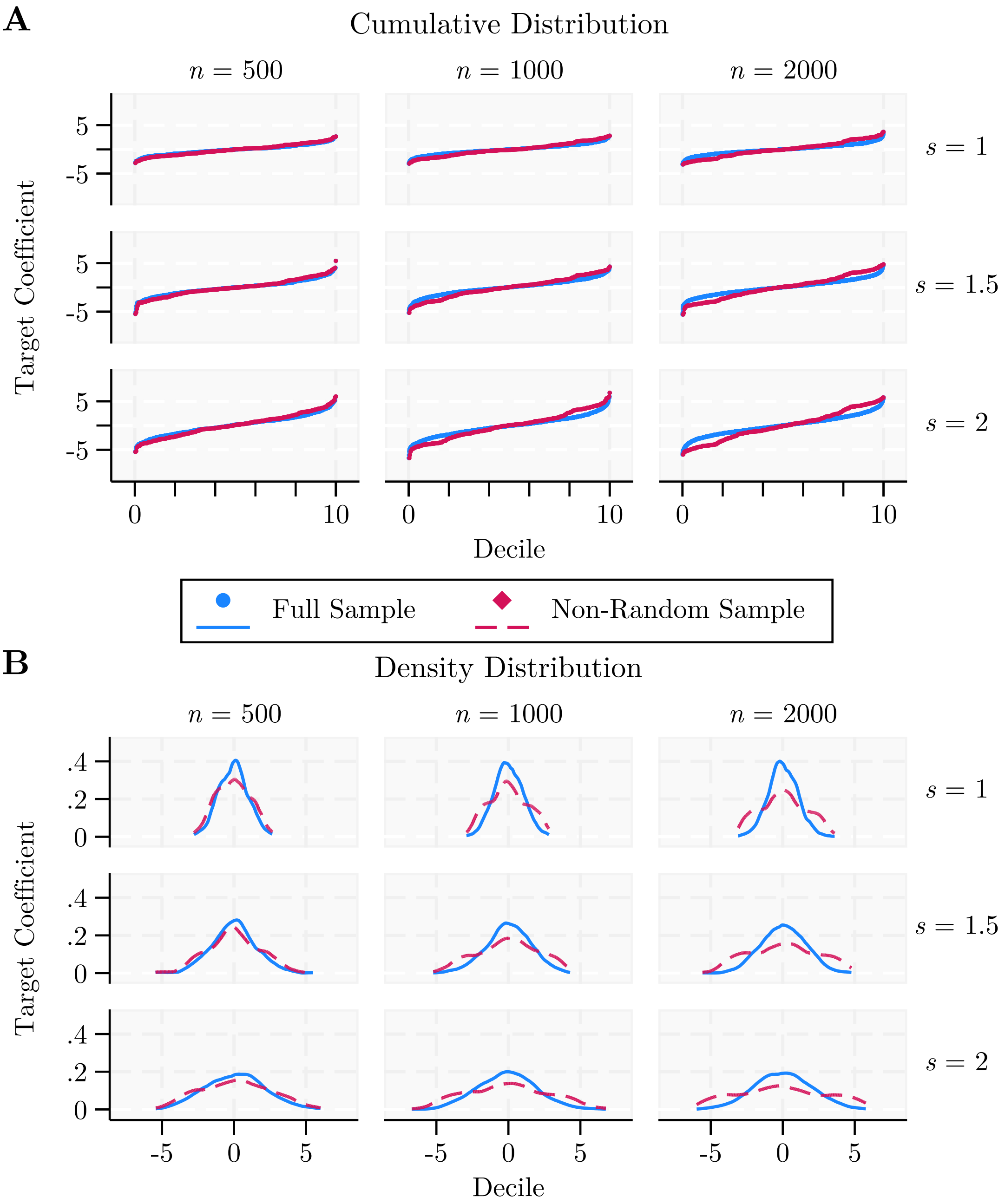

Figure 1: **Distortion Due to Non-Random Sampling.** Simulated data plotted as cumulative outcomes curves (Panel A) and density distributions (Panel B)

Two points are noteworthy. First, non-random sampling is most problematic in large multiverses with wide modeling distributions. Consider the plot in the upper left of Panel A: Here, the multiverse is small

($n = 500$) and has a narrow distribution ($\sigma = 1$). The visual representation of the results is virtually identical whether all estimates or a non-random subset are plotted. Now move from left to right. As the size of the multiverse increases—that is, as more mid-sized estimates are being excluded—the non-random sample begins to fan out, diverging more and more from the full sample. While differences are small at first, they grow quickly and can be significant in multiverses of millions or even billions of estimates [2]. To assess the role of variance, scan each column from top to bottom. For any given sample size, the gap between the full and sampled distributions widens as the variance of the modeling distribution increases. Thus, in the lower right of Panel A, where the effects of sample size and variance compound ($n = 2000$, $\sigma = 2$), visual distortion is most pronounced.

Second, non-random sampling can mislead researchers about the true causes of variation in multiverse results. Consider the graphs in Panel B, which revisit the simulated multiverse distributions from Panel A, this time plottted as density graphs. The impact of non-random sampling is clear: Because of the sampling strategy, the true, unimodal distribution of multiverse results morphs into a multi-model distribution (e.g., bottom right of Panel B). Normally, local models in the modeling distribution indicate the presence of a strongly influential model ingredient, such as an important control variable [3]. In this case, what is influential is the method of sampling estimates before graphing them.

Appendix B. Influence regressions: WLS vs. OLS.

Multiverse plots feature influence coefficients (to the right of the bottom panel) that quantify how much the target coefficient changes on average when switching from one parameter value to a reasonable alternative. These influence coefficients come from a two step regression which is closely related to classical meta-regression analysis [6].

Suppose we are interested in the effect of x on y, which is captured by β. Let $M = M_1, \ldots, M_J$ denote the set of all reasonable model specifications ($j = 1, \ldots, J$). For simplicity, consider a linear model where analysts disagree about the correct subset of K available control variables. In the first step, we estimate for each M_j:

$$y_j = \alpha_j + \hat{\beta}_j x_j + \sum_{k=1}^{K_j} \hat{\gamma}_{jk} z_{jk} + \epsilon_j \tag{1}$$

where α_j is the intercept, $\hat{\beta}_j$ is the estimated effect of x on y (i.e., the target coefficient), z_{jk} represents the k-th control variable in M_j, $\hat{\gamma}_{jk}$ is the corresponding coefficient, K_j is the number of control variables in M_j, and ϵ_j the error term. The resulting vector $\hat{\boldsymbol{\beta}} = (\hat{\beta}_1, \hat{\beta}_2, \ldots, \hat{\beta}_J)$ encompasses all estimates of β from the multiverse.

In the second step, we regress $\hat{\beta}_j$ on the subset of control variables in M_j. This meta-regression can be expressed as:

$$\hat{\beta}_j = \delta + \sum_{k=1}^{K} \theta_k d_{jk} + \nu_j \tag{2}$$

where δ is the intercept (i.e., the expected value of $\hat{\beta}_j$ when no controls are included), d_{jk} is a binary indicator that equals 1 if the k-th control variable is included in M_j, θ_k represents the marginal effect of including the k-th control variable on $\hat{\beta}_j$, and ν_j is the model-specific error term. To assess how influential the inclusion of a control variable is, examine θ_k. This coefficient quantifies how much the target coefficient changes on average if z_k is included as a predictor. By comparing θ_k across control variables $k = 1, ..., K$, we can determine which controls have the most influence on the target coefficient and how robust the estimated effect of x is to changes in model specification.

While we believe that this framework is promising for identifying sensitive researcher decisions, three potential pitfalls deserve attention. First, parameter influence may be non-linear. Including z_1 as a control variable, for instance, might influence the target coefficient *only* if z_2 is also included in the model. In this case, the influence regression is misspecified and requires the interaction term $z_1 * z_2$. The same applies to quadratic effects and higher order interactions. Since such influence interactions are hardly intuitive, researchers may turn to indicators on model fit of equation 2 for guidance whether a simple linear combination of model parameters predicts $\hat{\beta}_j$ sufficiently well.

Second, equation 2 should be estimated using weighted-least-squares (WLS). Like in meta-regression analysis [6], the dependent variable in equation 2 is itself an estimate. As such, it comes with varying precision (e.g., due to different sample sizes across multiverse models). Because $\hat{\beta}_j$ has varying precision, ν_j cannot be assumed to be independent and identically distributed [6]. There is heteroskedasticity and OLS is no longer the best linear unbiased estimator [4]. Conveniently, in multiverse analysis there is full information on the form of heteroskedasticity through SE_j. WLS uses a variation of equation 2 where each estimate $\hat{\beta}_j$ is weighted with its squared precision [6].

Third, all estimates in the influence regression must be on the same scale. This point may seem obvious, but warrants careful attention. Consider a scenario where researchers disagree on whether to use a linear probability model (LPM) or a logistic regression. Since these functional forms produce coefficients on different scales, their estimates must be converted into comparable units [3]. While this conversion is simple for LPM vs. logit, it can be challenging or even impossible for more complex functional forms. Moreover, the use of WLS mandates to also convert the standard errors. If analysts convert $\hat{\beta}_j$ but not SE_j, WLS will produce nonsensical results.

In the hurricane example, scaling is an issue. The multiverse suggested by Simonsohn et al. [1] contains coefficients from both negative binomial and log-transformed linear OLS models, which produce estimates on different scales. In the negative binomial model, the target coefficient (i.e., the difference in predicted casualties for female vs. male hurricanes) is measured in absolute deaths. In the log-transformed linear model, the difference in predicted values resides on the log-scale and requires re-transformation for comparison. This entails exponentiating the predictions for female and male hurricanes, calculating the difference, and adjusting the result with a "smearing estimate" [4]. However, obtaining meaningful standard errors for this difference is not straightforward. Because the hurricane example serves only illustrative purposes, we resort to OLS for estimating its influence regression.

References

[1] Simonsohn U, Simmons JP, Nelson LD. Specification Curve Analysis. Nature Human Behaviour. 2020;4(11):1208–1214. doi:10.1038/s41562-020-0912-z.

[2] Muñoz J, Young C. We Ran 9 Billion Regressions: Eliminating False Positives through Computational Model Robustness. Sociological Methodology. 2018-08;48(1):1–33. doi:10.1177/0081175018777988.

[3] Young C, Cumberworth E. Multiverse Analysis: Computational Methods for Robust Results. Cambridge University Press; 2025.

[4] Wooldridge JM. Introductory Econometrics: A Modern Approach. Seventh edition ed. Cengage Learning; 2020.

[5] Leamer E, Leonard H. Reporting the Fragility of Regression Estimates. The Review of Economics and Statistics. 1983-05;65(2):306. doi:10.2307/1924497.

[6] Stanley TD, Doucouliagos H. Meta-Regression Analysis in Economics and Business. Routledge; 2012.
